# Nucleolin facilitates nuclear retention of an ultraconserved region containing *TRA2β4* and accelerates colon cancer cell growth

**DOI:** 10.18632/oncotarget.25510

**Published:** 2018-06-01

**Authors:** Yuzuru Satake, Yuki Kuwano, Tatsuya Nishikawa, Kinuyo Fujita, Saki Saijo, Miki Itai, Hiroki Tanaka, Kensei Nishida, Kazuhito Rokutan

**Affiliations:** ^1^ Department of Pathophysiology, Institute of Biomedical Sciences, Tokushima University Graduate School, Tokushima 770–8503, Japan

**Keywords:** nucleolin, transcribed-UCR, TRA2β4, colon cancer, cell growth

## Abstract

Transcribed-ultraconserved regions (T-UCRs), which contain conserved sequences with 100% identity across human, rat and mouse species, are a novel category of functional RNAs. The human *transformer 2β* gene (*TRA2B*) encodes a UCR that spans exon 2 (276 bp) and its neighboring introns. Among five spliced RNA variants (*TRA2β1-5*) transcribed from the *TRA2B* gene, only *TRA2β4* contains the conserved exon 2. *TRA2β4* is overexpressed in colon cancer cells and accelerates cell growth by blocking the transcription of *CDKN1A*. However, the mechanisms underlying the overexpression of *TRA2β4* in colon cancer cells are unknown. Using biotinylated RNA pull-down assays followed by liquid chromatography-mass spectrometric analysis, we identified nucleolin as a *TRA2β4*-binding protein. Knockdown of nucleolin reduced the nuclear retention of *TRA2β4* and accelerated its degradation in the cytoplasm, whereas nucleolin overexpression increased *TRA2β4* levels and its mitogenic activity. Nucleolin directly bound to *TRA2β4* exon 2 via the glycine/arginine-rich (GAR) domain. Overexpression of GAR-deficient nucleolin failed to increase *TRA2β4* expression and growth of colon cancer cells. RNA fluorescence *in situ* hybridization showed that *TRA2β4* co-localized with nucleolin in nuclei but not with the mutant lacking GAR. Our results suggest that specific interactions between nucleolin and UCR-containing *TRA2β4* may be associated with abnormal growth of colon cancer cells.

## INTRODUCTION

Transformer 2β (Tra2β) belongs to the serine/arginine-rich (SR)-like protein splicing factor family. It contains two SR domains and an RNA recognition motif (RRM), and it functions as a sequence-specific pre-mRNA splicing enhancer [1, 2]. Overexpression of Tra2β protein is associated with several types of cancer, including those in breast, ovary, and colon. It appears to accelerate tumor growth and metastasis, and induces drug resistance [3–5]. The human *TRA2B* gene consists of 10 exons and 9 introns, and produces five mRNA isoforms (*TRA2β1* to *5*) through alternative splicing [6]. A functional full-length Tra2β protein is translated from *TRA2β1* mRNA lacking exon 2, a region that encodes multiple premature termination codons (PTCs) [6]. The *TRA2β4* mRNA isoform (referred to here as *TRA2β4*) contains exon 2 and is not translated to a functional protein [6]. Aberrant splicing occurs in many genetic and acquired diseases, including cancer [7–10]. PTC-containing mRNA splice variants are usually degraded through nonsense-mediated mRNA decay (NMD). However, we previously reported that Hu antigen R regulated alternative splicing of *TRA2β* pre-mRNA to selectively produce *TRA2β4* in human colon cancer cells under oxidative stress [11]. In addition, transcribed *TRA2β4* was retained preferentially within the nucleus and was resistant to RNA surveillance NMD [12]. The accumulated *TRA2β4* in the nucleus regulates gene expression (including *CDKN1A*) by occupying transcriptional factor Sp1, resulting in the promotion of cell growth by interrupting p21-related cellular senescence [12].

**Figure 1:**
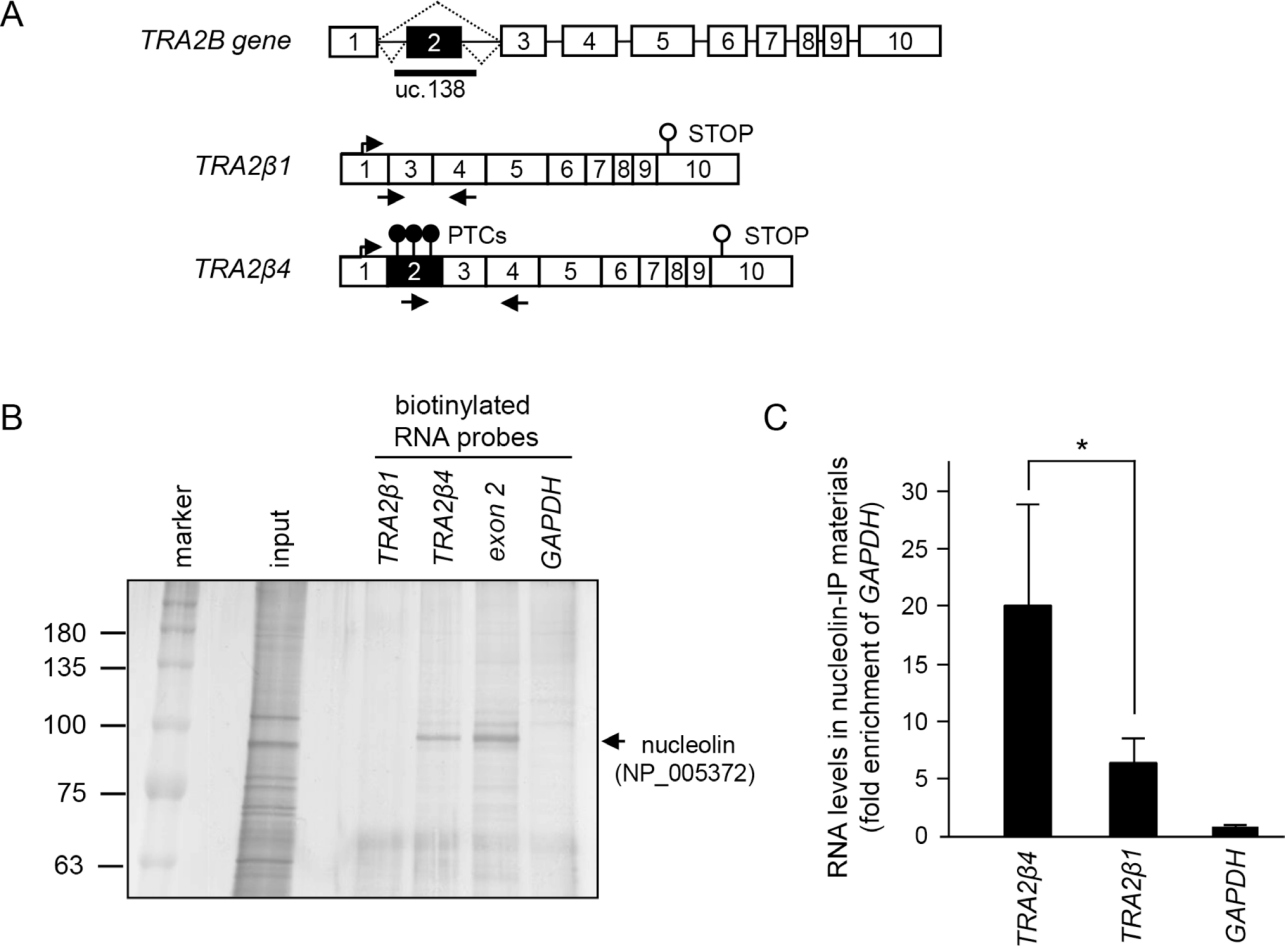
*TRA2β4* interacts with nucleolin (**A**) Schematic diagram of the human *TRA2B* gene. Exons are indicated by open boxes and Arabic numbers. Filled boxes denote the ultraconserved exon 2. Two major splice variants *TRA2β1* and *TRA2β4* are generated from the *TRA2B* gene and the use of each exon is shown. Black arrows show the specific primers used to detect each of the transcripts. PTCs; premature stop codons. (**B**) After biotinylated RNA pull-down assays using biotinylated exon 2 probes, the purified proteins were resolved by SDS-PAGE and visualized by silver staining. According to the analysis of the separated ~100 kDa protein by mass spectrometry, nucleolin (NP_005372) was one of RNA-binding proteins in the precipitated materials. (**C**) Cell lysates from HCT116 were subjected to a RIP assay using an anti-nucleolin antibody. Immunoprecipitated RNAs were quantified by qPCR. Data are shown as enrichment relative to *GAPDH*. Values are means ± s.d. (*n* = 6). ^*^Significantly different by unpaired Student's *t*-test (*p* < 0.05).

Intriguingly, the human *TRA2B* gene contains a 419-bp genomic segment with perfect human-to-rodent sequence identity, a segment termed the “ultraconserved region” (UCR) [13]. This UCR spans exon 2 (276 bp) and its neighboring introns. Among the five isoforms generated from *TRA2B*, only *TRA2β4* contains a complete version of exon 2 (Figure 1A) [6]. There are 481 described UCRs, and more than half of them do not encode a protein [14]. However, 68% of UCRs are transcribed, a group that constitutes a novel category of functional RNAs, transcribed-UCRs (T-UCRs) [14]. Genome-wide expression profiling revealed the distinct signatures of T-UCRs in human leukemia and carcinomas and an association with tumorigenesis [15]. Importantly, the expression levels of several T-UCRs show a tissue-specific pattern that is altered in human diseases, especially in cancer [12, 16–20]. However, understanding of the regulatory mechanisms in T-UCR expression remains unclear.

Recently, several lines of evidence have suggested the important role of RNA-binding proteins (RBPs) in the regulation of the functional activities of long non-coding RNAs (lncRNAs) [21, 22]. For example, the bromodomain protein Brd4 regulates a lncRNA called *HOTAIR*, which is essential for glioblastoma proliferation [23]. Accordingly, we focused on the association between RBPs and a UCR-containing *TRA2β4* in the nucleus and examined whether post-transcriptional regulation impacted aberrant expression of T-UCR.

Distinct functional non-coding RNAs retained within the mammalian cell nucleus are now referred to as “nuclear-retained regulatory RNAs”, and they are suggested to play structural roles or act as riboregulators [24, 25]. Although the majority of lncRNAs reside in the nucleus, only a portion have been functionally characterized. In this study, using biotinylated RNA pull-down assays followed by liquid chromatographic-mass spectrometric (LS/MS) analysis, we identified nucleolin as a *TRA2β4*-binding protein. Nucleolin is a multifunctional protein that is located primarily in the nucleus. It plays significant roles in the metabolism and function of RNA, including transcription, ribosome assembly, mRNA turnover and translation, and it participates in the regulation of cell fate [26–28]. By interacting with target mRNAs that typically contain AU-rich and/or G-rich elements, nucleolin regulates their expression state [27, 29]. We suggest here a potential role of nucleolin in nuclear localization of *TRA2β4* that facilitates abnormal growth of T-UCR-bearing cancer cells.

## RESULTS

### Association between *TRA2β4* and nucleolin

The human *TRA2B* gene contains an ultraconserved region (uc.138) that spans exon 2 and its neighboring introns. The *TRA2β* mRNA isoform that contains exon 2 (*TRA2β4*) is produced by alternative splicing (Figure 1A). Although exon 2 contains multiple PTCs, *TRA2β4* resides preferentially in the nuclei of HCT116 cells and could escape from the NMD surveillance pathway [12]. To reveal the mechanism supporting the expression of *TRA2β4* in colon cancer cells, we first searched for nuclear RNA-binding protein(s) that specifically interacted with and retained *TRA2β4* in nuclei. Here, we employed biotinylated RNA pull-down analyses with the biotinylated exon 2 probe followed by LS/MS analysis. The isolated proteins with molecular masses around 100 kDa preferentially contained RNA-binding proteins including nucleolin and splicing factors, such as heterogeneous nuclear ribonucleoprotein (hnRNP) UL 1, hnRNPR, hnRNPU, and hnRNPQ (Figure 1B and Supplementary Table 1). These hnRNPs likely participate in alternative splicing reaction of exon 2. We were particularly interested in nucleolin (NP_005372), a nuclear protein related to cell survival, proliferation, and invasion of cancer cells [30]. To confirm the binding between nucleolin and endogenous *TRA2β4*, ribonucleoprotein immunoprecipitation (RIP) analysis was carried out with an anti-nucleolin antibody and specific primers for *TRA2B* transcripts. *TRA2β4* was more abundantly precipitated in nucleolin-IP materials compared with *TRA2β1* (Figure 1C). Taken together, *TRA2β4* was likely associated with nucleolin in the nucleus of HCT116 cells.

### Identification of nucleolin-binding sites in *TRA2β4*

**Figure 2:**
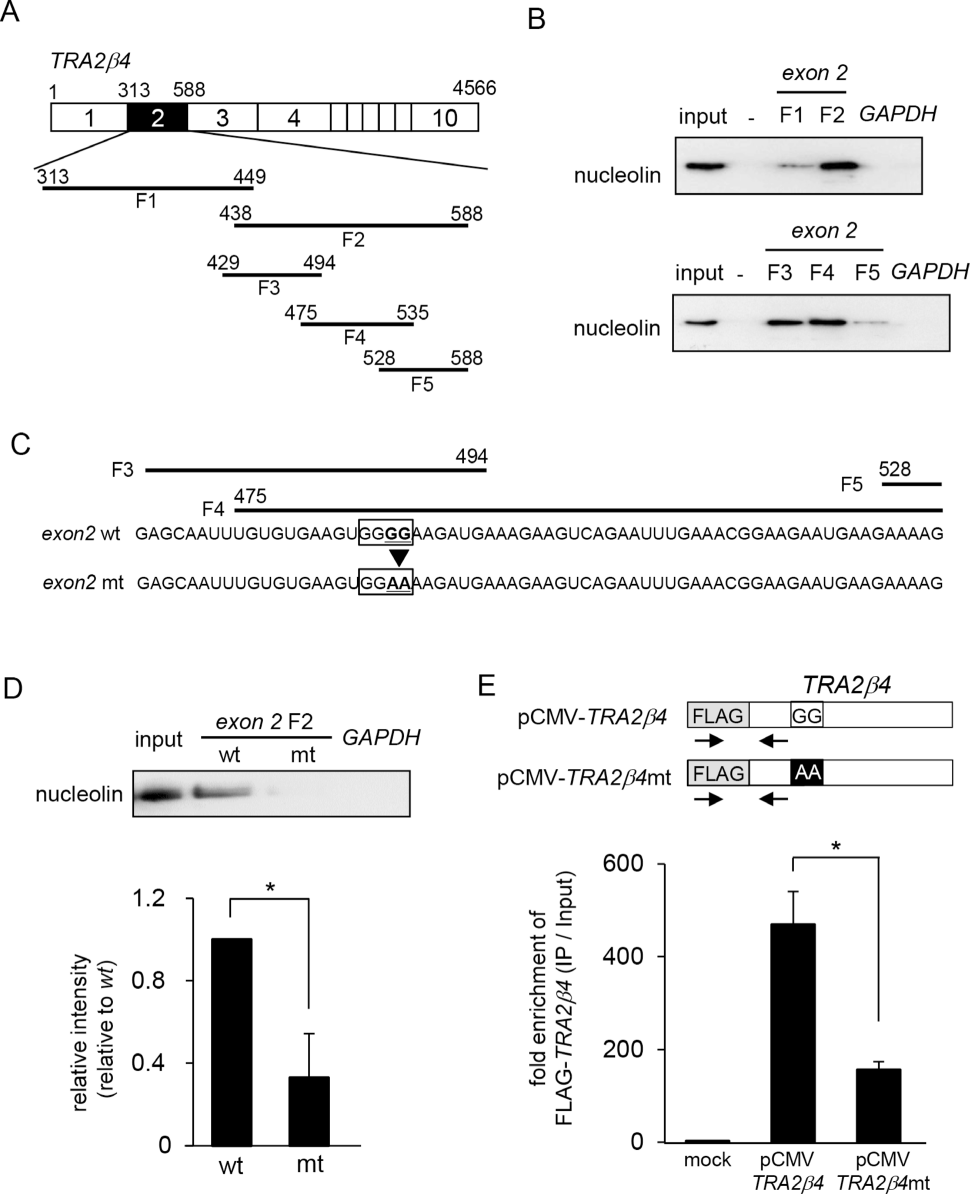
Identification of nucleolin-binding sites in *TRA2β4* (**A**) Schema of the fragments of *TRA2β4* that were used for *in vitro* binding assays. (**B**) A biotinylated RNA pull-down assay was carried out using lysates prepared from HCT116 cells and the biotinylated RNA fragments. (**C**) The RNA sequence in exon 2 F3 to F5. The introduction of two-point mutations (mt; 485-GGGG-488 to 485-GGAA-488) in *TRA2β4* exon 2. (**D**) A biotinylated RNA pull-down assay was carried out using biotinylated RNA fragments with (mt) or without (wt) mutations. (**E**) The constructs coding FLAG-tagged full length *TRA2β4* (pCMV- *TRA2β4*) or the mutated *TRA2β4* (pCMV- *TRA2β4* mt) were generated. These plasmids were transfected into HCT116 cells and then RIP assays were carried out with an anti-nucleolin antibody. The exogenous *TRA2β4* in IP materials was detected using the primers shown as arrows. Each value represents the mean ± s.d. from 3 independent experiments. ^*^Significantly different by unpaired Student's *t*-test (*p* < 0.05).

To determine the binding sites of *TRA2β4* exon 2 that were responsible for its association with nucleolin, we prepared a series of biotinylated RNA fragments (F1- F5) that encoded various lengths of *TRA2B* exon 2 (Figure 2A). Biotinylated RNA pull-down assays showed more specific association of nucleolin with fragment F2 compared with that of F1 (Figure 2B, upper panel). Then, fragment F2 was further divided into three parts (F3-F5). The putative binding site(s) was likely present in both F3 and F4 (Figure 2B, lower panel). As shown in Figure 2C, the common sequences between F3 and F4 (475–494) contain a G-rich element (485-GGGG-488), which was reported to be essential for binding with nucleolin [31]. The introduction of two-point mutations (mt; 485-GGGG-488 to 485-GGAA-488) into exon 2 F2 significantly reduced its interaction with nucleolin (Figure 2D). We also generated constructs encoding FLAG-tagged full length *TRA2β4* (pCMV- *TRA2β4*) or two point-mutated *TRA2β4* (pCMV- *TRA2β4* mt) (Figure 2E). These plasmids were transfected into HCT116 cells and then RIP assays were carried out with an anti-nucleolin antibody. Using primer sets to detect endogenous FLAG-tagged *TRA2β4*, we confirmed that mutagenesis in exon 2 decreased the interaction between *TRA2β4* and nucleolin (Figure 2E).

### *TRA2β4* recognition domain in nucleolin

**Figure 3:**
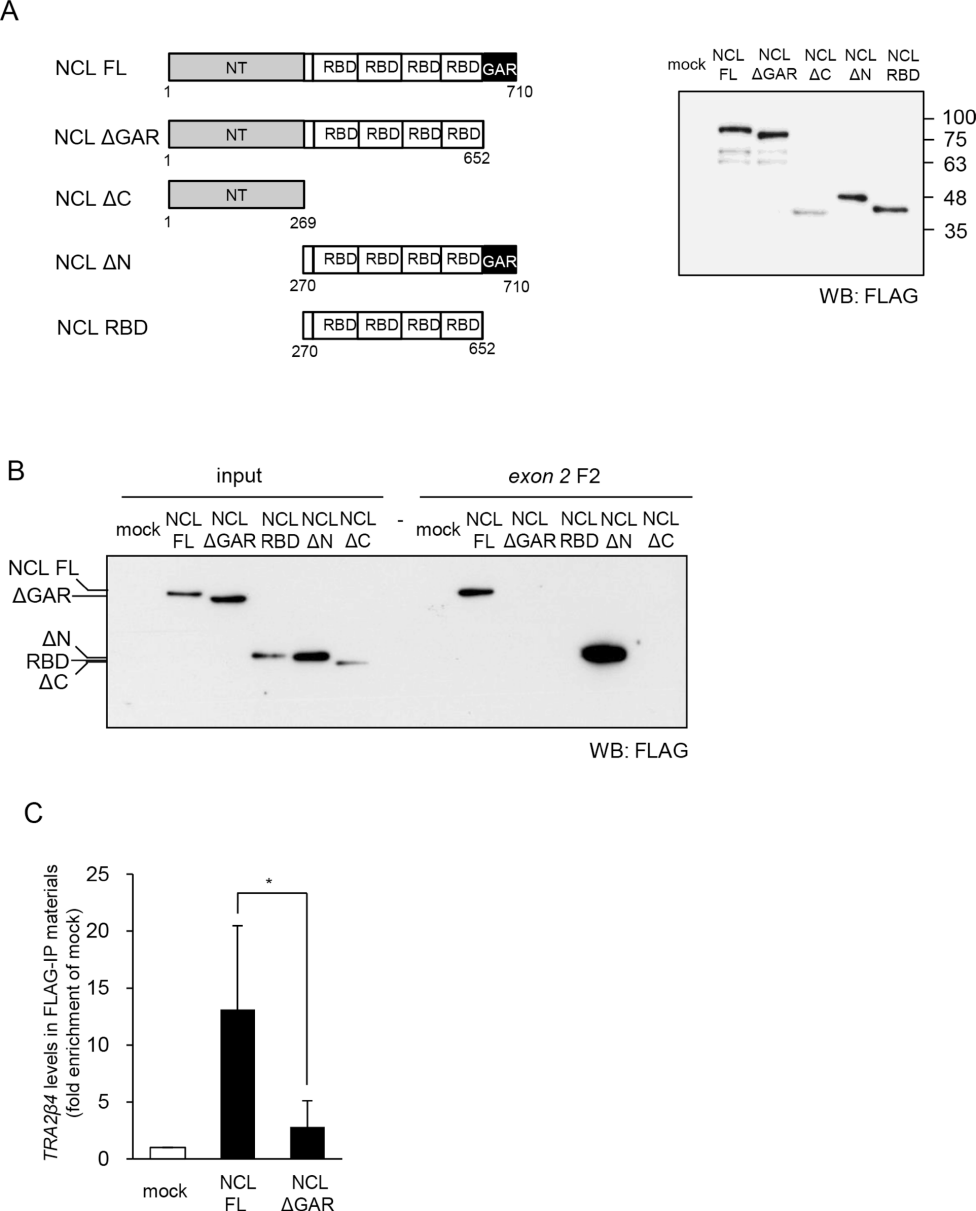
Characterization of *TRA2β4* recognition domain in nucleolin (**A**) The scheme of constructs encoding full-length nucleolin (NCL FL), lacking the GAR (NCL ΔGAR), lacking the C-terminus (NCL ΔC), lacking the N-terminal domain (NT) (NCL ΔN), and RNA-binding domains (RBDs) only (NCL RBD). Each plasmid was transfected into HCT116 cells for 48 hours, and then whole-cell lysates were subjected to Western blotting using an anti-FLAG antibody. (**B**) A biotinylated RNA pull-down assay was carried out using biotinylated RNA fragment F2 and lysates prepared from HCT116 cells that were transfected with each plasmid. The bound nucleolin was detected by Western blotting using anti-FLAG antibody. (**C**) The plasmids bearing full-length or GAR-deficient nucleolin were transfected into HCT116 cells and then a RIP assay was carried out with an anti-FLAG antibody. *TRA2β4* in IP materials was detected by qPCR. Each value represents the mean ± s.d. from 3 independent experiments. ^*^Significantly different by unpaired Student's *t*-test (*p* < 0.05).

To further verify the specific association between nucleolin and *TRA2β4*, we generated a construct encoding FLAG-tagged nucleolin. Nucleolin includes 3 major domains: an acidic N-terminal region (NT), RNA binding domains (RBDs), and a glycine/arginine-rich (GAR) domain (Figure 3A). Nucleolin binds to the pre-ribosomal RNA processing complex through the NT (1–269 aa). The central region of nucleolin contains four tandem RRMs (307–647 aa), an RBD, which mediates the association with mRNAs. An arginine-glycine-glycine (RGG) repeat in the GAR domain (649–698 aa) in the C-terminal region of nucleolin plays an important role for interaction with target mRNAs as well as other proteins [32].

We first produced 5 different constructs expressing full-length or truncated nucleolin proteins: (1) pCMV-FLAG-tagged full-length nucleolin (NCL FL), (2) nucleolin lacking GAR (NCL ΔGAR), (3) NT only (NCL ΔC), (4) RBDs and GAR (NCL ΔN), and (5) RBDs (NCL RBD) (Figure 3A). Cell lysates were prepared from HCT116 cells overexpressing one of these constructs and subjected to pull-down assays using biotinylated exon 2 F2. As shown in Figure 3B, full-length nucleolin was detected in the complex with biotinylated exon 2 using an anti-FLAG antibody. The association of nucleolin with exon 2 did not require its N-terminal domain nor tandem RBDs of nucleolin, whereas the presence of the c-terminal domain seemed to be essential for the association with *TRA2β4* exon 2 (Figure 3B). Finally, we confirmed that the association between nucleolin and *TRA2β4* exon 2 disappeared when GAR was deleted from nucleolin (Figure 3B). To further validate the association, we asked whether the deletion of GAR affected the interaction between nucleolin and endogenous *TRA2β4*. Using an anti-FLAG antibody, RIP was employed to detect the association between *TRA2β4* and either wild-type or GAR-deficient nucleolin. The RIP assay showed that the GAR-deleted nucleolin mutant could not bind to *TRA2β4* (Figure 3C).

### Reduction of *TRA2β4* levels after nucleolin knockdown

**Figure 4:**
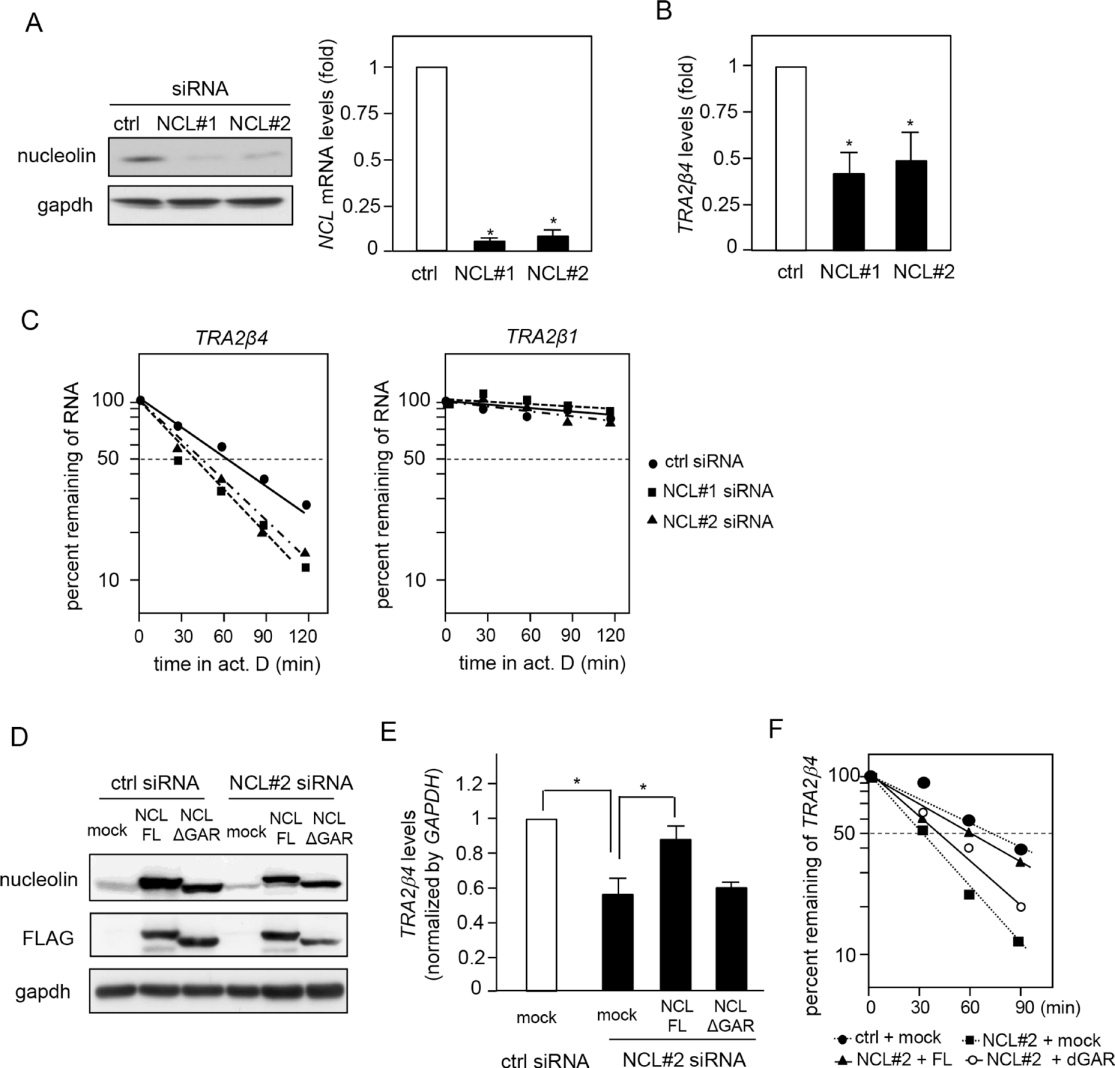
Effect of nucleolin on *TRA2β4* stability (**A**) HCT116 cells were treated with siRNAs for 48 h as follows: 10 nM control, NCL#1 (targeting the coding region of nucleolin), or NCL #2 (targeting 3′-UTR). Nucleolin levels were analyzed by Western blotting and qPCR. (**B**) The levels of *TRA2β4* in control or nucleolin-silenced cells were measured by qPCR. (**C**) HCT116 cells were transfected with control, NCL #1, or NCL #2 siRNAs for 48 h and then incubated in the presence of 2 μg/mL actinomycin D for the indicated times. *TRA2β1* and *TRA2β4* levels were measured by qPCR and plotted on a logarithmic scale to calculate the time required for each RNA to reach one-half of its initial abundance (50%, dashed line). (**D**) After downregulation of endogenous nucleolin using an siRNA targeting its 3′-UTR (NCL#2), nucleolin expression was rescued by transfection with plasmids encoding a FLAG-tagged coding region of nucleolin with or without GAR. (**E**) The levels of *TRA2β4* in nucleolin-rescued cells were measured by qPCR. (**F**) *TRA2β4* levels in nucleolin-rescued cells after incubation with 2 μg/mL actinomycin D for the indicated times were measured by qPCR. The results were plotted on a logarithmic scale to calculate the time required for each RNA to reach one-half of its initial abundance (50%, dashed line).

We next examined whether the specific interaction between *TRA2β4* and nucleolin affected *TRA2β4* expression. A reduction of nucleolin levels using siRNAs targeting the coding region (NCL #1) or 3′-UTR (NCL #2) of *NCL* mRNA significantly decreased *TRA2β4* levels in HCT116 cells (Figure 4A and 4B). To elucidate the mechanisms for the *TRA2β4* decline following nucleolin knockdown, we measured *TRA2β4* stability in control or nucleolin siRNA-transfected cells. Nucleolin knockdown significantly accelerated the degradation rate of *TRA2β4*, whereas it did not affect the stability of *TRA2β1* mRNA (Figure 4C). After downregulation of endogenous nucleolin using NCL #2 siRNA targeting its 3′-UTR, nucleolin expression was rescued by transfection with plasmids encoding a FLAG-tagged coding region of nucleolin with or without GAR (Figure 4D). Overexpression of full-length nucleolin completely rescued *TRA2β4* expression, whereas GAR-deficient nucleolin had no effect on *TRA2β4* levels (Figure 4E). We also confirmed that the replacement of the full-length, but not GAR-deficient nucleolin, blocked the accelerated degradation of *TRA2β4* in HCT116 cells treated with the nucleolin 3′-UTR-targeting siRNA (Figure 4F). Hence, we hypothesize that nucleolin may influence *TRA2β4* stability via association through the GAR domain.

### Regulation of nuclear localization of *TRA2β4* by nucleolin

**Figure 5:**
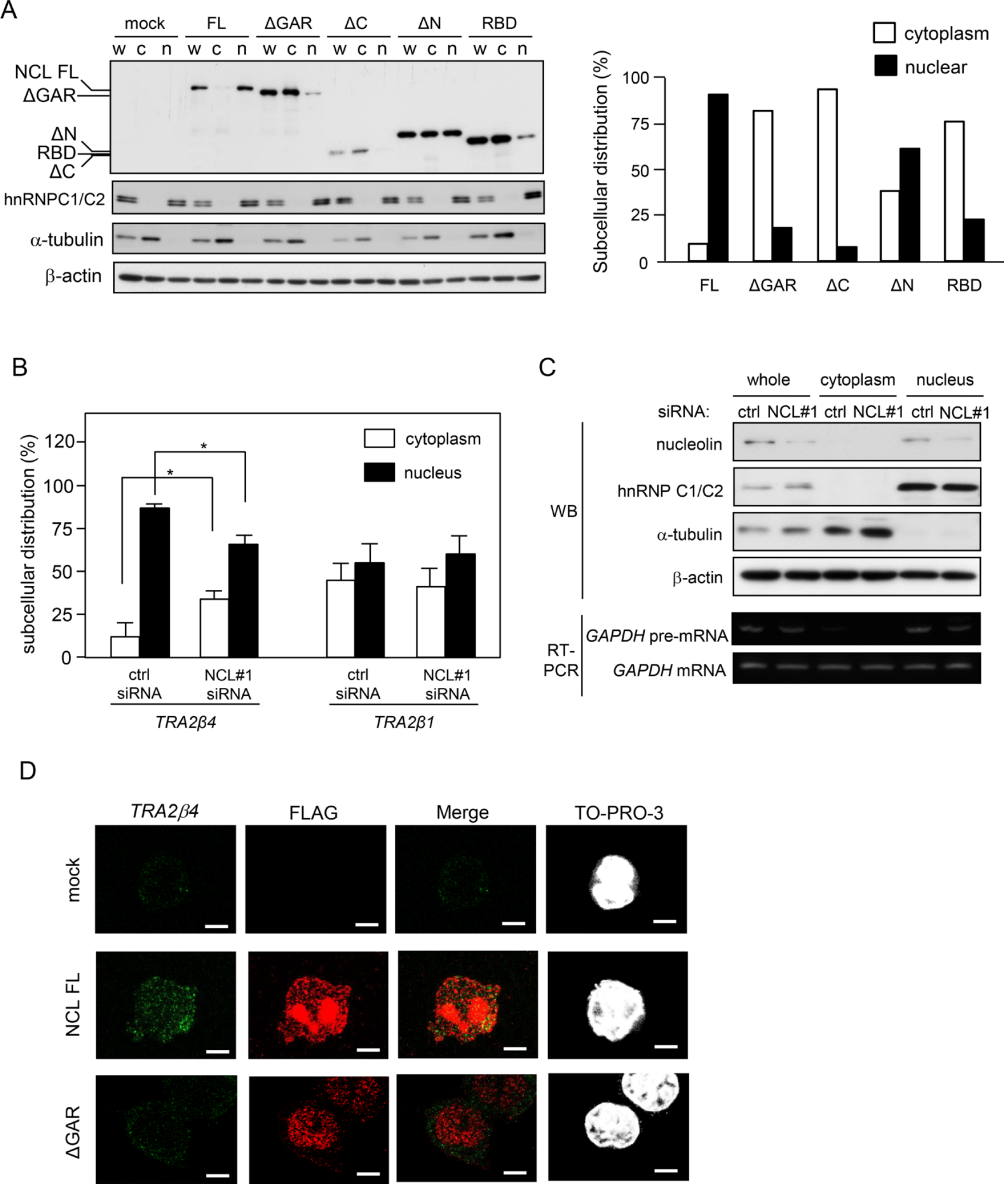
Nucleolin is essential for nuclear localization of *TRA2β4* (**A**) After transfection with the truncated nucleolin, the subcellular localization was measured by Western blotting. FLAG signals were quantified by densitometry. (**B**) After NMD was inhibited with cycloheximide treatment (100 μg/mL), the amounts of *TRA2β4* and *TRA2β1* mRNAs in the cytoplasmic or nuclear fraction were analyzed by qPCR. (**C**) Purities of the extracted nuclear and cytoplasmic fractions were confirmed by Western blotting using antibodies for cytosolic (α-tubulin) or nuclear proteins (hnRNP C1/C2) and by RT-PCR using primers targeting *GAPDH* pre-mRNA as a nuclear marker. (**D**) After a 24-h transfection of full-length or ΔGAR nucleolin, subcellular localization of *TRA2β4* (green) and nucleolin (red) was examined by RNA-fluorescence *in situ* hybridization (RNA-FISH) using a probe that specifically hybridized to exon 2 (313-588 nt) and anti-FLAG antibody. Nucleoli were counterstained with TO-PRO-3. Scale bars, 5 μM.

To test whether nucleolin regulated the nuclear localization of *TRA2β4*, we first examined the subcellular localization of nucleolin. We prepared HCT116 cells that overexpressed FLAG-tagged full-length nucleolin, or one of the truncated nucleolins (Figure 3A). As shown in Figure 5A, full-length nucleolin mainly resided in nuclei, whereas the deletion of GAR (ΔGAR, ΔC, RBD) led to nucleolin's loss of its nuclear retention ability. After NMD was inactivated by treatment with 100 μg/mL cycloheximide, we measured the amounts of *TRA2β4* and *TRA2β1* mRNAs in the cytoplasmic or nuclear fraction. As shown in Figure 5B, silencing of nucleolin decreased the nuclear content of *TRA2β4* without changing the subcellular localization of *TRA2β1* mRNA. The purities of the extracted nuclear and cytoplasmic fractions were confirmed by Western blotting using antibodies for cytosolic (α-tubulin) or nuclear proteins (hnRNP C1/C2) and by RT-PCR using primers targeting *GAPDH* pre-mRNA as a nuclear marker (Figure 5C).

In a previous study, we demonstrated that *TRA2β4* was predominantly located in the nucleic fraction compared with *TRA2β1* mRNA [12]. In this study, RNA FISH staining for *TRA2β4* was employed with immunostaining of FLAG-tagged full-length nucleolin or ΔGAR overexpressed in HCT116 cells. *TRA2β4* signals were detectable in the nucleus of mock-transfected, control cells (Figure 5D, upper panel). Overexpression of full-length nucleolin increased *TRA2β4* signals in the nucleus (Figure 5D, middle panel), and the overexpressed nucleolin partially co-localized with *TRA2β4*. However, in ΔGAR-overexpressing cells, *TRA2β4* signals were diffusely distributed both in the nucleus and the cytoplasm. ΔGAR signals did not co-localize with *TRA2β4* signals (Figure 5D, lower panels).

### Roles of nucleolin and *TRA2β4* in cell growth and resistance to anticancer drug

**Figure 6:**
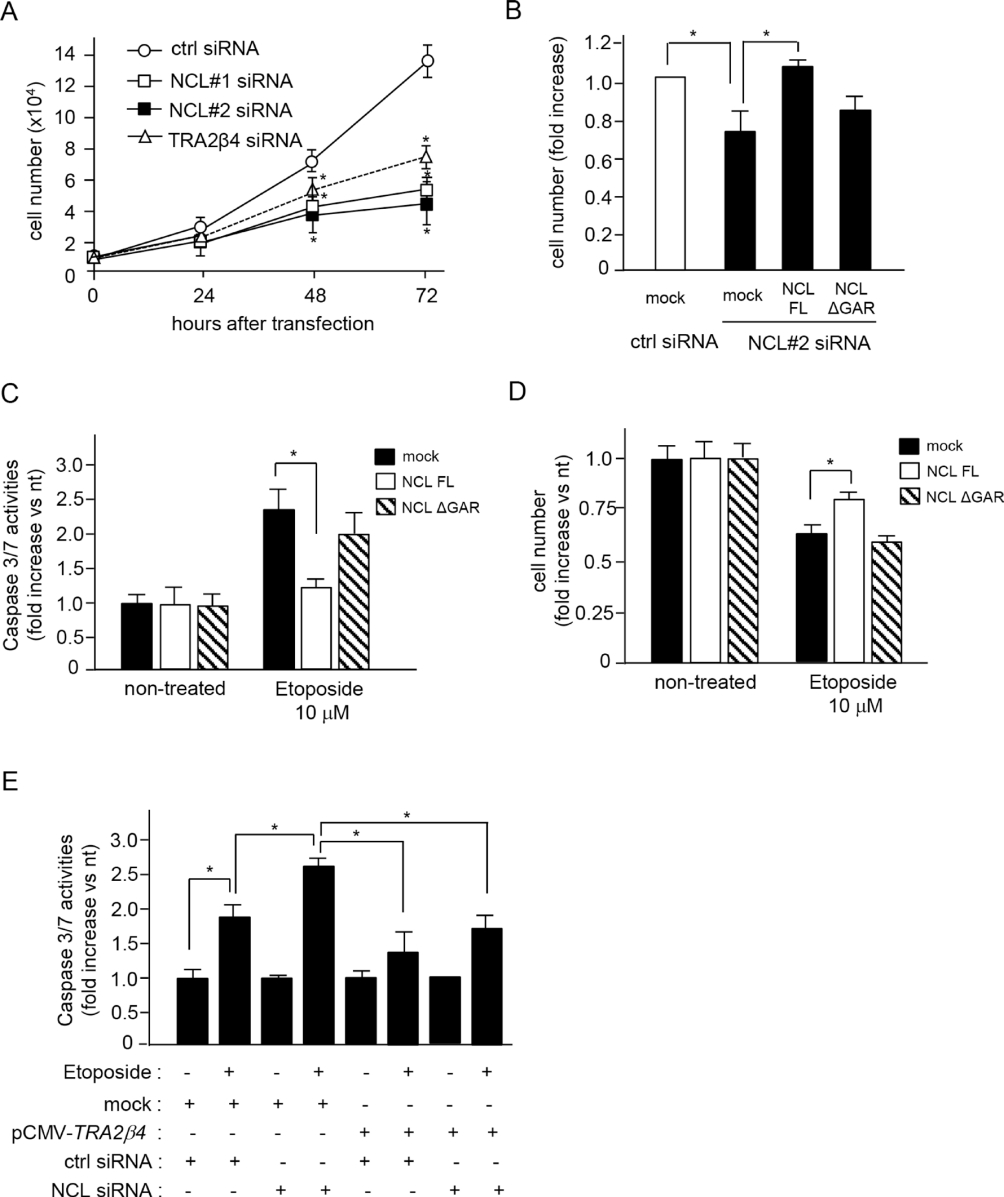
Association between nucleolin and *TRA2β4* is essential for cell growth and susceptibility to an anticancer drug (**A**) HCT116 cells (1 × 10^4^ cells) were seeded in 35-mm dishes and transfected with the indicated siRNA. Subsequently, growing cells were harvested and counted at the indicated times. Values are means ± s.d. from 4 independent experiments. ^*^Significantly different by unpaired Student's *t*-test compared with control siRNA-treated cells (*p* < 0.05). (**B**) After transfection with control (ctrl) or NCL siRNA #2 for 12 h, the cells were treated with plasmids encoding a FLAG-tagged full-length or ΔGAR nucleolin. After a 48-h transfection, these cells were harvested and counted. Values are means ± s.d. from 4 independent experiments. ^*^Significantly different by unpaired Student's *t*-test (*p* < 0.05). (**C**, **D**) HCT116 cells were treated with the indicated siRNAs and plasmids for 24 h, and then cells were exposed to 10 μM etoposide for 24 h. Subsequently, growing cells were harvested and counted or used for measurement of caspase 3/7 activities. Values are means ± s.d. from 4 independent experiments. ^*^Significantly different by unpaired Student's *t*-test (*p* < 0.05). (**E**) After HCT116 cells were treated with the indicated siRNAs and plasmids for 24 h, the cells were exposed to 10 μM etoposide for 24 h. Subsequently, growing cells were harvested and caspase 3/7 activities were measured. Values are means ± s.d. from 3 independent experiments. ^*^Significantly different by unpaired Student's *t*-test (*p* < 0.05).

Several reports have shown that the inhibition of nucleolin with siRNAs or anti-nucleolin agents inhibits cell growth [33–36]. In fact, knockdown of nucleolin with NCL siRNA #1 and #2 significantly inhibited HCT116 cell growth (Figure 6A). The growth inhibition with NCL siRNA #2, which targeted 3′-UTR of *NLC* mRNA, was reversed by overexpression of a full-length nucleolin, but not by that of a GAR deficient nucleolin (ΔGAR) (Figure 6B). Knockdown of nucleolin induces apoptosis via caspase 3 activation in response to oxidative stress or anticancer agents such as doxorubicin and etoposide [37, 38]. When HCT116 cells were treated with NCL siRNA #2 and exposed to etoposide for 24 h, caspases 3/7 were activated (Figure 6C) and cell numbers were decreased (Figure 6D). In these endogenous nucleolin-silenced cells, overexpression of full-length nucleolin, but not that of ΔGAR, could block the activation of caspases 3/7 (Figure 6C) and rescue the etoposide-induced cell loss (Figure 6D). To further confirm the importance of the nucleolin-mediated *TRA2β4* expression for the resistance to etoposide, we rescued *TRA2β4* in nucleolin knockdown cells. As shown in Figure 6E, overexpression of *TRA2β4* significantly inhibited the etoposide-induced activation of caspases 3/7 in nucleolin knockdown cells. These results suggested that nucleolin seemed to be necessary for the tumorigenic property of *TRA2β4*.

As previously reported [12], *TRA2β4* siRNA significantly inhibited growth of HCT116 cells (Supplementary Figure 1A, 1B and Figure 6A) without changing either protein or mRNA levels of nucleolin as well as Tra2β (Supplementary Figure 1C–1E). In the previous study, we found that *TRA2β4* accelerated cell growth by downregulating p21 expression through an inhibition of Sp1-binding to the *CDKN1A* promoter [12]. We therefore tested whether nucleolin modulated the *TRA2β4*/p21 pathway. Chromatin immunoprecipitation (ChIP) assays indicated that nucleolin did not directly associate with the *p21/CDKN1A* promoter (Supplementary Figure 2A). In addition, silencing of Sp1 had no effect on nucleolin binding to the *CDKN1A* promoter, suggesting that nucleolin did not compete with Sp1 for binding to the promoter of *CDKN1A* (Supplementary Figure 2A). The reduction of endogenous nucleolin with NCL siRNA #2 caused the decline of *TRA2β4* levels (Figure 4E) and resulted in a corresponding increase of *p21/CDKN1A* mRNA levels in mock-transfected, control cells (Supplementary Figure 2B). Overexpression of full length nucleolin, but not ΔGAR, could block this increase of *p21/CDKN1A* mRNA levels (Supplementary Figure 2B). These results suggest that nucleolin may play an important role in the *TRA2β4*/p21-mediated regulation of cell growth. The GAR domain may be crucial for this function.

### Gene expression signatures in *TRA2β4*- or nucleolin-silenced cells

**Figure 7:**
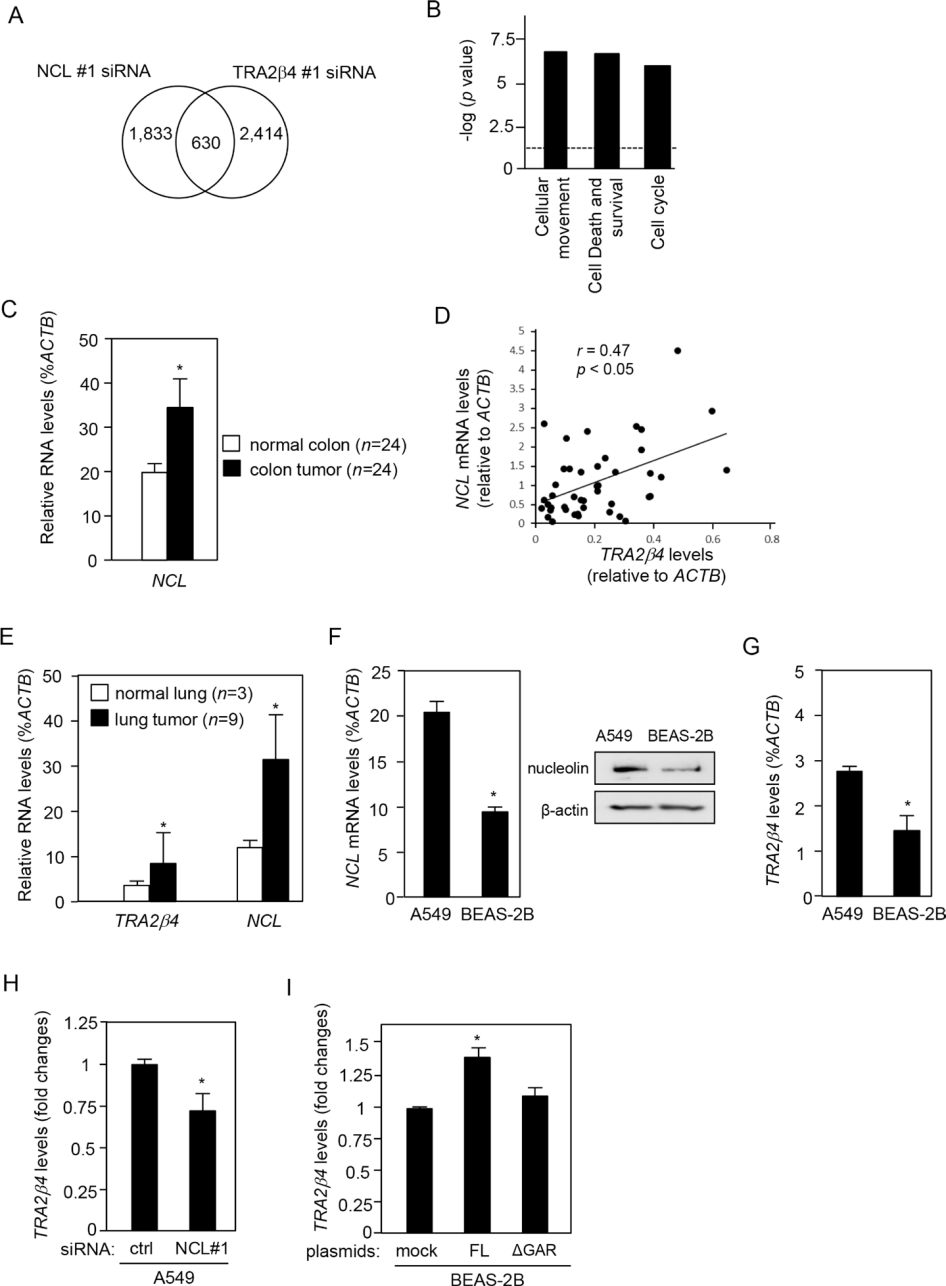
Nucleolin regulates *TRA2β4* expression in colon and breast cancer cells (**A**) Microarray analysis showed that NCL siRNA #1-treated cells differentially expressed 2,493 genes (≥ 1.5-fold), compared with control siRNA-treated cells. *TRA2β4*-silenced cells altered the expression of 3,044 genes (≥ 1.5-fold). A total of 630 genes were commonly changed in the same direction between *TRA2β4-* and nucleolin-silenced cells. (**B**) Commonly regulated 630 genes were subjected to Ingenuity Pathway Analysis (QIAGEN Bioinformatics) to identify biologically relevant functions. (**C**) Using colon adenocarcinoma TissueScan Tissue qPCR arrays (HCRT103, OriGene), *NCL* and *ACTB* mRNAs were measured by qPCR. *ACTB* mRNA was used as an endogenous quality control. ^*^Significantly different by paired Student's *t*-test (*p* < 0.05). (**D**) The correlation between *TRA2β4* and *NCL* expression in a colon cancer cDNA array was analyzed by determining the Pearson's correlation coefficient. (**E**) Expression levels of *TRA2β4*, *NCL*, and *ACTB* mRNAs were measured by qPCR using lung cancer cDNA libraries (CSRT101, OriGene). *ACTB* mRNA was used as an endogenous quality control. Values are means ± s.d. ^*^Significantly different by unpaired Student's *t*-test (*p* < 0.05). (**F**) Both protein and mRNA levels of nucleolin in lung carcinoma (A549) and lung epithelial cells (BEAS-2B) were analyzed by qPCR or Western blotting. Values are means ± s.d. from 3 independent experiments. ^*^Significantly different by unpaired Student's *t*-test (*p* < 0.05). (**G**) The levels of *TRA2β4* expression were measured by qPCR in A549 and BEAS-2B cells. Values are means ± s.d. from 4 independent experiments. ^*^Significantly different by unpaired Student's *t*-test (*p* < 0.05). (**H**) After A549 cells were treated with 10 nM of the indicated siRNA for 48 h, changes in the expression levels of *TRA2β4* were measured by qPCR. *GAPDH* mRNA was used as an endogenous quality control. Values are means ± s.d. from 4 independent experiments. ^*^Significantly different by unpaired Student's *t*-test compared with control (ctrl) siRNA-treated cells (*p* < 0.05). (**I**) After BEAS-2B cells were transfected with mock or plasmids encoding nucleolin with/without GAR for 48 h, changes in the expression level of *TRA2β4* were measured by qPCR. *GAPDH* mRNA was used as an endogenous quality control. Values are means ± s.d. from 3 independent experiments. ^*^Significantly different by unpaired Student's *t*-test compared with mock-transfected cells (*p* < 0.05).

Finally, gene expression signatures in *TRA2β4-* or nucleolin-silenced cells were examined to understand the underlying molecular mechanism and to assess the ability of nucleolin to impact nuclear-retained *TRA2β4*-mediated gene expression (NCBI Gene Expression Omnibus #GSE112758). As shown in Figure 7A, microarray analysis showed that NCL siRNA #1-treated cells differentially expressed 2,493 genes (≥ 1.5-fold), compared with control siRNA-treated cells. *TRA2β4*-silenced cells altered the expression of 3,044 genes (≥ 1.5-fold). Interestingly, a total of 630 genes were commonly changed in the same direction between *TRA2β4-* and nucleolin-silenced cells. Ingenuity Pathway Analysis (QIAGEN Bioinformatics) was employed to identify biological functions relevant to the 630 genes. As shown in Figure 7B, the enriched molecular and cellular functions of genes regulated in both nucleolin- and *TRA2β4*-silenced cells included 1) Cellular Movement (*p* = 1.35E-07), 2) Cell Death and Survival (*p* = 3.92E-07), and Cell Cycle (*p* = 2.00E-06). Moreover, the top disease associated with the 630 genes was Cancer (*p* = 1.88E-12). These results suggest that *TRA2β4* may be one of the important targets for facilitating abnormal cell growth.

### Nucleolin-dependent expression of *TRA2β4* in colon and lung cancer cells

Using a paired colon cancer cDNA panel prepared from 24 patients (HCRT103; OriGene, Rockville, MD, USA), we measured *NCL* mRNA levels. Compared with paired normal colon tissues, colon cancers expressed significantly higher levels of *NCL* mRNA (Figure 7C). *TRA2β4* was overexpressed in human colon cancer cell lines and colon carcinomas [12]. We examined the association between the expression of *NCL* mRNA and that of *TRA2β4*, and we found that the *TRA2β4* levels were positively correlated with those of *NCL* in both normal and colon cancer tissues (*r* = 0.47, Pearson's correlation coefficient) (Figure 7D).

We also examined the expression of *TRA2β4* and *NCL* mRNA in lung cancer cells. Expression levels of *TRA2β4* and *NCL* mRNAs were upregulated in lung cancer cDNA libraries (CSRT101, OriGene) compared with normal lung tissues (Figure 7E). Human lung cancer cell line A549 cells expressed higher amounts of *NCL* mRNA and nucleolin protein than those in lung epithelial BEAS-2B cells (Figure 7F). At the same time, A549 cells expressed significantly higher amounts of *TRA2β4* compared with BEAS-2B cells (Figure 7G). Nucleolin knockdown also decreased in *TRA2β4* levels in A549 cells (Figure 7H). In contrast, overexpression of full-length (but not GAR-deficient nucleolin) in BEAS-2B cells significantly increased *TRA2β4* expression (Figure 7I). Thus, the nucleolin-mediated regulation of *TRA2β4* expression was not unique to HCT116 colon cancer cells.

## DISCUSSION

About 70% of the 481 UCRs in the human genome are transcribed and appear to be functional RNAs, T-UCRs [13]. For each T-UCR, one corresponding to the sense genomic sequence is named ultraconserved (Uc.) ‘+’, and the other complementary sequence is Uc. ‘-’. Recent studies have shown that T-UCRs function as regulators in multiple pathways such as pri-miRNA processing, transcriptional regulation, translation, and chromatin modification [20, 39, 40]. Dysregulated expression of distinct T-UCRs may be directly linked to the development of various diseases. For instance, decreased expression of Uc.173 in the hippocampus is associated with lead-induced neuronal apoptosis by inter-regulation of miRNAs [41]. Uc.261 is overexpressed in intestinal mucosa of active Crohn's disease and is suggested to participate in the damage to tight junctions in inflammation [42]. In addition, differential expression of T-UCRs has been observed in several types of cancer [14, 16, 18, 19]. Uc.190, Uc.233 and Uc.270 levels are increased in human pancreatic adenocarcinoma and knockdown of each of them reduced the proliferation of MIA PaCa-2 pancreatic cancer cells [19]. Uc. 8+ works as a natural decoy for miR-596 and cooperates in the promotion and development of bladder cancer [20]. Carotenuto *et al.* reported that Uc. 158- was activated by the Wnt/β-catenin pathway in liver cancer and drove cellular growth and migration, possibly by modulating miR-193b expression [18]. One of 5 transcripts generated from the human *TRA2B* gene, *TRA2β4* is categorized as a T-UCR (Uc.138+). *TRA2β4* is overexpressed in human gastric cancer cells and colon cancer cells and facilitates their growth [11, 12, 43]. Knockdown of *TRA2β4* in colon cancer cells leads to cellular senescence through p21 induction, meaning that *TRA2β4* is one of the key molecules that causes aberrant cell cycle regulation of cancer cells [12].

The molecular mechanisms of T-UCR expression are not fully understood. Several studies have demonstrated that T-UCR expression is altered at the transcriptional level in cancer cells. A batch of T-UCRs (Uc.160+, Uc.285+, Uc.346+) undergo specific CpG island hypermethylation-associated silencing in colon cancer cells [44]. Suppression of Uc.160+ expression by DNA hypermethylation was also observed in human gastric cancer cells [17]. Tumor-related activation of transcription factor Egr1 caused induction of genes encoding T-UCRs in pancreatic adenocarcinoma [19]. Recent studies have suggested that functional RNAs, including lncRNAs, are also regulated in a post-transcriptional manner. LncRNAs are reported to exert their function by various mechanisms, such as interacting with regulatory proteins to modulate their processing and metabolism and directing localization within cellular compartments. A novel lncRNA, colorectal neoplasia differentially expressed (*CRNDE*), promotes cell proliferation and metastasis of colorectal carcinoma [45]. The turnover of *CRNDE* is dependent on its associating protein, heterogeneous nuclear ribonucleoprotein U-like 2 (hnRNPUL2) [45]. On the other hand, it is not fully understood whether RNA-binding proteins are involved in the regulation of T-UCR expression at the post-transcriptional levels. In colon cancer cells, *TRA2β4* preferentially resides in the nucleus and could escape RNA degradation in the cytoplasm [12]. Non-coding RNAs retained within the nucleus are referred to as nuclear-retained regulatory RNAs, and their structural roles are recognized as riboregulators [46]. To elucidate the mechanism for nuclear retention of *TRA2β4,* we characterized *TRA2β4*-associated nuclear proteins.

This study shows for the first time that a nuclear RNA-binding protein, nucleolin, is a possible regulator of *TRA2β4* expression. Nucleolin interacted with *TRA2β4* via a G-rich sequence (485-GGGG-488) in exon 2. This interaction is likely crucial for nuclear retention and the resultant stabilization of *TRA2β4*. Overexpression of full-length nucleolin significantly increased the stability of *TRA2β4* and accelerated cell proliferation. A significant part of overexpressed nucleolin co-localized with *TRA2β4* in the nucleus. In contrast, nucleolin knockdown decreased *TRA2β4* levels by accelerating its degradation. Nucleolin is composed of three main structural domains: the N-terminus, the central region, and the C-terminal domain [26]. The C-terminus is the arginine/glycine-rich (GAR) domain and interacts with nucleic acids [28]. Importantly, GAR-deficient nucleolin did not modulate nuclear localization of *TRA2β4*, resulting in accelerated decay of *TRA2β4* in the cytoplasm. GAR-deficient nucleolin could not induce *TRA2β4*-mediated tumorigenic effects such as accelerated cell growth and anticancer drug resistance. Finally, we confirmed the positive correlation between *TRA2β4* and nucleolin expression levels in colon cancer cells.

Nucleolin is a clinically important protein as it possesses oncogenic properties. In fact, nucleolin is overexpressed in a number of malignant tumors, including cancers of the breast, lung, stomach, pancreas, cervix, prostate as well as colorectal cancers, melanomas and leukemias [28, 47–52]. For instance, nucleolin binds to a receptor tyrosine kinase (ErbB2) and promotes its activation, resulting in enhancement of tumorigenicity of breast cancer [52]. In gastric cancer, Yang *et al*. demonstrated that nucleolin mediated the epithelial-mesenchymal transition via upregulation of the Erk1/2 and Akt pathways [50]. Moreover, nucleolin stabilizes the mRNA of anti-apoptotic protein bcl-2 [53]. Recent studies have proposed that nucleolin is a novel target for anticancer therapy as demonstrated by the effects of antagonistic molecules [33, 54, 55]. Nucleolin is a multifunctional protein that is mainly found in the nucleolus, but it is also observed in the nucleoplasm and cytoplasm. Nucleoplasmic nucleolin is involved in the regulation of translation and stability of oncogenic mRNAs [33, 56]. Interestingly, nucleolin may protect cancer cells from senescence by controlling intracellular localization of the telomerase complex [57].

In the previous study, we showed that *TRA2β4* accelerated cancer cell growth by downregulating p21 expression through an inhibition of Sp1 binding to the *CDKN1A* promoter [12]. In this study, we found that nucleolin did not compete with Sp1 for binding to the promoter of *CDKN1A*. We also assessed the ability of nucleolin to impact nuclear-retained *TRA2β4*-mediated gene expression indirectly by measuring changes in gene expression in nucleolin- or *TRA2β4*-silenced cells. Both manipulated cells commonly changed expression of a large number of genes (630 genes). IPA ranked “Cancer” as the top disease associated with the 630 genes. Of course, nucleolin exerts its oncogenic properties by interacting with many targets as described above. *TRA2β4* may be one of the important targets for facilitating abnormal growth of cancer cells. The GAR-domain may be essential for the activity of tumorigenic T-UCR *TRA2β4* retained within the nucleus. Further studies are needed to reveal the pathological significance of the nucleolin-mediated aberrant expression of *TRA2β4*.

## MATERIALS AND METHODS

### Cell culture and transfection

Cells of the human colorectal carcinoma line (HCT116) were cultured in McCoy's 5A medium (Thermo Fisher Scientific, Waltham, MA) supplemented with 5% (v/v) heat-inactivated fetal bovine serum (FBS) and antibiotics (penicillin and streptomycin). Human lung cell lines (BEAS-2B and A549) were maintained in RPMI-1640 medium (Thermo Fisher Scientific) supplemented with 10% FBS. These cells were cultured at 37° C in 5% CO_2_. Twenty-four h prior to transfection, 1 × 10^4^ cells were cultured in 6-well plates and then treated with 10 nM control or nucleolin (*NCL*) mRNA-targeting siRNA (Santa Cruz, CA, USA) using Lipofectamine RNAiMAX (Thermo Fisher Scientific). Expression plasmids encoding the full-length or truncated nucleolin were transfected with Lipofectamine 3000 according to the manufacturer's protocol (Thermo Fisher Scientific).

Apoptosis was assessed by measuring caspase-3/7 activities using the caspase-Glo 3/7 assay kit (Promega, Fitchburg, WI, USA) according to the manufacturer's protocol.

### Western blotting

Whole cell lysates were prepared using RIPA (Thermo Fisher Scientific) with a complete protease inhibitor cocktail (Roche, Mannheim, Germany). After HCT116 cells were incubated in cytosolic lysis buffer (10 mM Tris-HCl, pH 7.4, containing 100 mM NaCl, 2.5 mM MgCl_2_, and 40 μg/ml digitonin) for 10 min at 4° C, the resultant lysates were centrifuged at 2,060 *g* for 8 min at 4° C, and the supernatants were collected as cytosolic extracts. The pellets were washed twice with the lysis buffer and then lysed with RIPA buffer. After centrifugation at 10,000 *g* for 10 min at 4° C, the resultant supernatants were collected as nuclear extracts, as previously described [12]. Ten micrograms of the extracted proteins were separated by sodium dodecyl sulfate (SDS)-polyacrylamide gel electrophoresis and then transferred to a polyvinylidene fluoride membrane (Bio-Rad, Hercules, CA, USA). After blocking with 5% nonfat dry milk (Cell Signaling Technology, Danvers, MA, USA) for 1 h at room temperature, the membrane was incubated with anti-nucleolin (C23) (Santa Cruz), anti-FLAG (Sigma-Aldrich, St. Louis, MO, USA), anti-hnRNPC1/C2 (Santa Cruz), anti-α-tubulin (Santa Cruz), anti-β-actin (Abcam, Cambridge, UK), or anti-glyceraldehyde 3-phosphate dehydrogenase (Gapdh) (Santa Cruz) antibody overnight at 4° C. Following incubation with an appropriate secondary antibody for 1 h at room temperature, the bound antibodies were detected with Pierce Western Blotting Substrate (Thermo Fisher Scientific). The intensities of the bound antibodies were quantified by using ImageJ software (National Institutes of Health, USA).

### Biotinylated RNA pull-down analysis

RNA fragments were generated by PCR using specific primers containing the T7 RNA polymerase promoter sequence (CTAATACG ACTCACTATAGGGAGA [T7]) (Supplementary Table 2). Biotinylated transcripts were synthesized *in vitro* using the MAXIscript Kit (Thermo Fisher Scientific) and the Biotin-11-CTP (PerkinElmer, CT, USA). The synthesized transcripts were then purified with ssDNA/RNA Clean and Concentrator (Zymo Research, CA, USA). After HCT116 cells were lysed with RIPA buffer, whole-cell lysates were prepared by centrifugation at 10,000 *g* for 10 min at 4° C and used for the biotinylated RNA pull-down assay. Eighty μg of whole-cell lysates were incubated with 2.5 μg of the biotinylated transcripts in TENT buffer (10 mM Tris-HCl buffer, pH 8.0, containing 1 mM EDTA, 250 mM NaCl, and 0.5% Triton X-100) for 30 min at room temperature [5, 11]. After adding Dynabeads M-280 Streptavidin (Thermo Fisher Scientific), the mixture was incubated for 1 h at room temperature. The bound proteins were pulled down using a magnetic particle separator and analyzed by Western blotting or liquid chromatography-mass spectrometry analysis (nanoLC-MS/MS, CapLC, Q-Tof).

### Real-time quantitative PCR (qPCR)

Total RNA was extracted from cells using the RNAiso Plus (Takara, Tokyo, Japan) and precipitated in the presence of Dr. GenTLE Precipitation Carrier (Takara). Isolated RNAs were reverse-transcribed using ReverTra Ace qPCR RT Master Mix (Toyobo, Osaka, Japan). *TRA2*β*1*, *TRA2β4*, *NCL*, *ACTB*, and *GAPDH* mRNA levels were measured using specific primer sets and SYBR Green Master Mix (Thermo Fisher Scientific) (Supplementary Table 2). SiRNA-treated cells were incubated with 2 μg/mL actinomycin D for the indicated times and then the amounts of *TRA2β4* and *GAPDH* mRNAs in the cells were measured by qPCR using 18S rRNA for normalization.

### Ribonucleoprotein immunoprecipitation (RIP) analysis

Twenty μl of Protein A Sepharose (Sigma-Aldrich) and 3 μg of the anti-nucleolin antibody were mixed in 1 ml of NT-2 buffer (50 mM Tris HCl; 150 mM NaCl; 1 mM MgCl_2_; 0.05% Nonidet P-40) [27]. The mixture was rotated overnight at 4° C and then washed 3 times with ice-cold NT-2 buffer. HCT116 cells were lysed with lysis buffer (pH 7.5) consisting of 25 mM Tris-HCl, 150 mM NaCl, 1 mM EDTA, 1% (v/v) Nonidet P-40, 5% (v/v) glycerol, and 100 U/ml RNase inhibitor (Nacalai Tesque, Tokyo, Japan) [11]. After these lysates were rotated for 2 h at 4° C with the pre-coated protein A Sepharose, they were treated with TURBO DNase (Thermo Fisher Scientific) for 10 min at 37° C, and then with 0.1% SDS and proteinase K solution (Wako, Osaka, Japan) for 10 min at 55° C. Finally, bound RNAs were extracted using a phenol-chloroform mixture and then subjected to qPCR.

### Plasmids

Full-length human *NCL* mRNA was cloned into the pCMV- FLAG vector (Agilent Technology, CA, USA). In brief, a fragment encoding a full-length nucleolin (pCMV-NCL FL; amino acid (aa) 1 to 710) was amplified by PCR using the following primer set: 5′-GGATCCATGGTGAAGCTCGCGAAGGCAGG-3′ (forward) and 5′-GAATTCCTATTCAAACTTCGTCTTCTTTCCT-3′ (reverse). The amplified fragment was subcloned into pCMV- FLAG vector using BamHI and EcoRI sites (these sequences are underlined). The truncated constructs were subcloned using the following primers: a construct for the deletion of GAR (pCMV-NCL ΔGAR; aa 1 to 652); 5′-GGATCCATGGTGAAGCTCGCGAAGGCAGG-3′ and 5′-GAATTCGCCACCTTCACCCTTAGGTTTGGC-3′; N-terminus of nucleolin (pCMV-NCL ΔC, aa 1 to 269), 5′-GGATCCATGGTGAAGCTCGCGAAGGCAGG-3′ and 5′-GAATTCCTCCTCCTCTTCTTCCTCCTCCTC A-3′; C-terminus of nucleolin (pCMV-NCL ΔN, aa 270 to 710), 5′-GGATCCGAAGAGCCTGTCAAA GAAGCACCT-3′ and 5′-GAATTCCTATTCAAACTTCG TCTTCTTTCCT-3′; RNA-binding domains (pCMV-NCL RBD; aa 270 to 652), 5′-GGATCCGAAGAGCCT GTCAAAGAAGCACCT-3′ and 5′-GAATTCGCCACC TTCACCCTTAGGTTTGGC-3′.

### RNA fluorescence *in situ* hybridization (RNA-FISH) and immunofluorescence staining

To detect *TRA2β4*, sequences complementary to exon 2 (313–588 nt) were transcribed *in vitro* using a FITC RNA labeling kit (Roche). HCT116 cells were fixed in 4% paraformaldehyde for 15 min and then permeabilized with 0.5% Triton X-100 for 15 min at room temperature. These cells were incubated with a 1 μg/mL FITC-labeled RNA probe in hybridization buffer (1 × Denhardt's solution containing 100 μg/mL tRNA, 0.01% Tween-20, and 5% dextran sulfate) at 55° C for 16 h. After washing, the cells were incubated overnight with anti-FITC polyclonal antibody (Thermo Fisher Scientific) and anti-FLAG monoclonal antibody (Sigma-Aldrich) at 4° C. After washing with TBST, the cells were treated with fluorophore-conjugated secondary antibodies (Alexa Fluor 488, 555: Thermo Fisher Scientific) for 1 h at room temperature and mounted with Vectashield (Vector Laboratories).

### Gene expression profiling

Total RNAs were extracted from cells using RNeasy kit (Qiagen) according to the manufacturer's protocol. Purified RNA quality was assessed by Agilent 2100 Bioanalyzer using an RNA 6000 Nano Labchip kit (Agilent Technologies, Santa Clara, CA, USA). RNA samples with 9.0 RNA integrity number (RIN) were used to measure mRNA expression profiles using a human mRNA microarray (SurePrint G3 Human; Agilent). The expression data were analyzed using GeneSpring 14.9 (Agilent).

### Statistical analysis

All statistical analyses were performed with the SPSS 21.0 software package (SPSS Inc., Chicago, IL, USA). Results are expressed as means ± SD. Significant differences between two groups were estimated by two-tailed Student's *t*-test. Non-parametric data were analyzed using a Wilcoxon-Mann-Whitney *U* test when comparing two groups. Pearson's correlation analysis was used to estimate the relationship between the expression levels of *NCL* and *TRA2β4*. ^*^p < 0.05 was considered statistically significant.

## SUPPLEMENTARY MATERIALS FIGURES AND TABLES





## Abbreviations

TRA2β: Transformer 2-beta
UCR: ultraconserved region
T-UCR: Transcribed-ultraconserved region
lncRNA: long noncoding RNA
qPCR: quantitative real-time polymerase chain reaction
