# Supplementary material for: Nucleolin facilitates nuclear retention of an ultraconserved region containing TRA2β4 and accelerates colon cancer cell growth
Source: Oncotarget. 2018 Jun 1;9(42):26817–33. doi: 10.18632/oncotarget.25510 (PMC6003563; doi:10.18632/oncotarget.25510)
Supplement: Supplementary file 1 [file oncotarget-09-26817-s001.pdf]

Nucleolin facilitates nuclear retention of an ultraconserved region containing *TRA2β4* and accelerates colon cancer cell growth

SUPPLEMENTARY MATERIALS

Supplementary Table 1: List of RNA-binding proteins determined by MS

Proteins	Symbol	Mass	Score	Molecular function
zinc finger CCCH-type antiviral protein 1	*ZC3HAV1*	101367	267	RNA-binding
heterogeneous nuclear ribonucleoprotein U-like 1	*hnRNPUL1*	95750	261	Activator, Repressor, Ribonucleoprotein, RNA-binding
heterogeneous nuclear ribonucleoprotein R	*hnRNPR*	70899	214	Ribonucleoprotein, RNA-binding
splicing factor proline/glutamine-rich	*SFPQ*	76141	212	Activator, DNA-binding, Repressor, RNA-binding
heterogeneous nuclear ribonucleoprotein Q	*hnRNPQ*	69590	109	Ribonucleoprotein, RNA-binding
heterogeneous nuclear ribonucleoprotein U	*hnRNPU*	88890	67	Activator, Chromatin regulator, DNA-binding, Repressor, Ribonucleoprotein, RNA-binding
nucleolin	*NCL*	76298	34	DNA-binding, RNA-binding
GPI-anchored protein p137	*CAPRIN1*	72707	34	Protein synthesis inhibitor, RNA-binding

Supplementary Table 2: Primer sets used for qPCR and *in vitro* transcription, and oligonucleotide sequences of siRNAs used. See Supplementary_Table_2

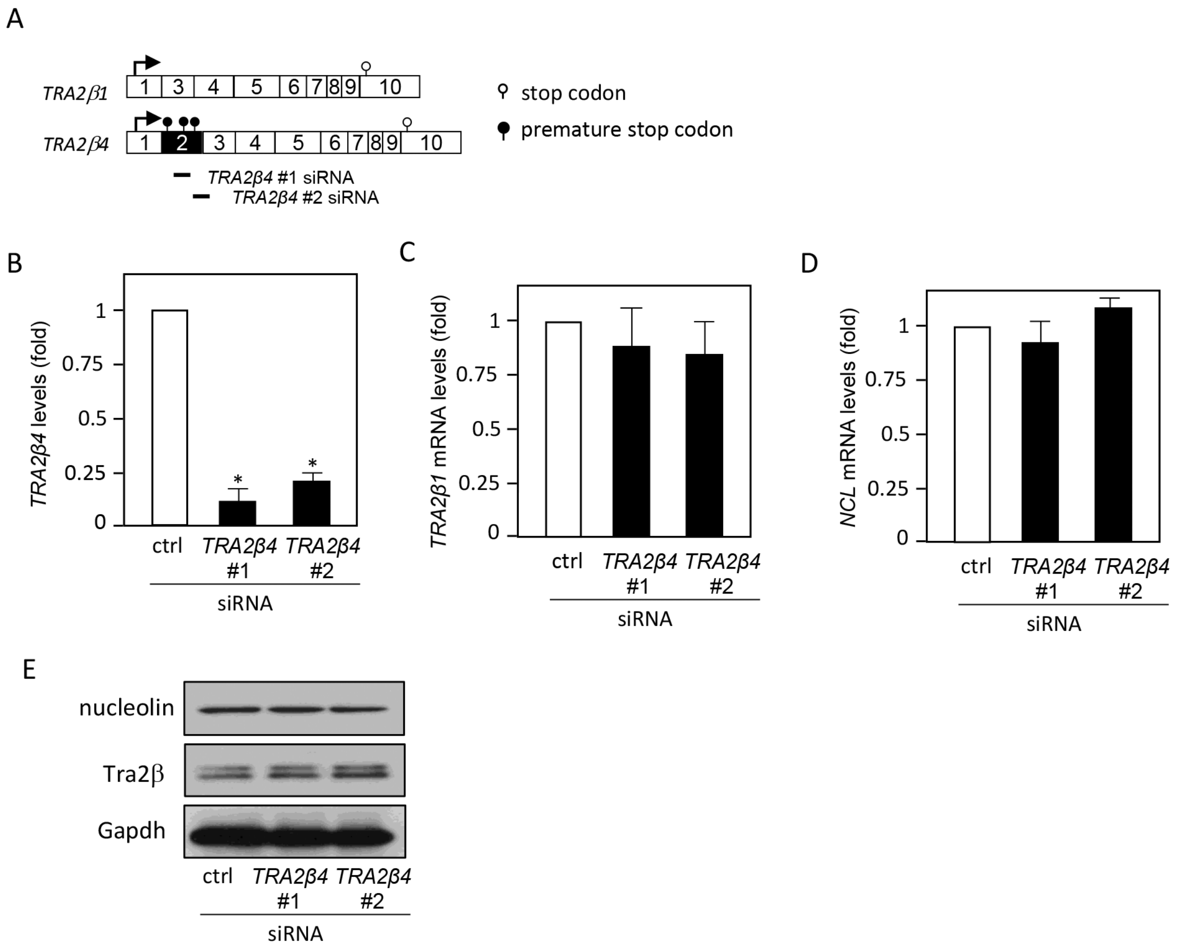

Supplementary Figure 1: Efficiency of *TRA2β4* siRNAs. (**A**) Two siRNAs targeting *TRA2β4* were designed in exon2 (Sequences are listed in Supplementary Table 2). (**B–D**) Ten nM of control or *TRA2β4* siRNAs were transfected into HCT116 cells for 48 hours. *TRA2β4, TRA2β1* and NCL mRNAs were measured by qPCR. *GAPDH* mRNA was used as an endogenous quality control. *Significantly different by unpaired Student's *t*-test ($p < 0.05$). (**E**) After whole-cell lysates were extracted from siRNA-treated cells, nucleolin, Tra2β, and Gapdh levels were analyzed by Western blotting.

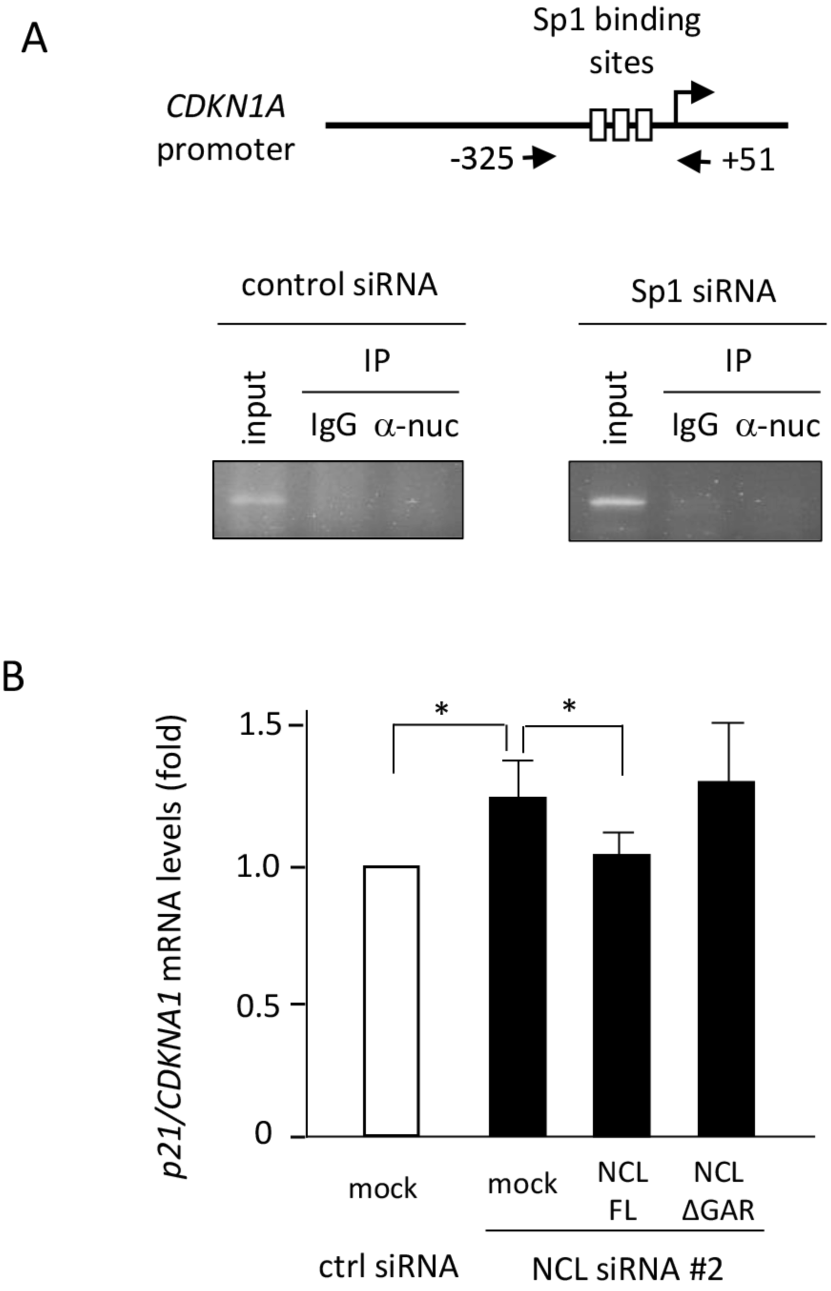

Supplementary Figure 2: Effect of nucleolin on p21 expression. (**A**) The association between nucleolin and the *p21/CDKN1A* promoter was measured by Chromatin immunoprecipitation (ChIP) assay using anti-nucleolin antibody (α-nuc). Normal rabbit IgG was used for a negative control (IgG). Black arrows show the specific primers used for ChIP assays. We previously reported that Sp1 bound to the −163/+31 bp-region containing the cluster of Sp1-binding sites [12]. ChIP assays showed that nucleolin did not associated with the *p21/CDKN1A* promoter. In addition, silencing of Sp1 had no effect on nucleolin binding to the promoter of *CDKN1A*. (**B**) After endogenous nucleolin was reduced by NCL siRNA #2, full length nucleolin or nucleolin lacking the GAR domain (ΔGAR) were transfected. Overexpression of full length nucleolin, but not ΔGAR, significantly reduced *CDKN1A* mRNA levels. *Significantly different by unpaired Student's *t*-test ($p < 0.05$).
