# Supplementary material for: Nucleolin facilitates nuclear retention of an ultraconserved region containing TRA2β4 and accelerates colon cancer cell growth
Source: Oncotarget. 2018 Jun 1;9(42):26817–33. doi: 10.18632/oncotarget.25510 (PMC6003563; doi:10.18632/oncotarget.25510)
Supplement: Supplementary file 2 [file oncotarget-09-26817-s002.docx]

**Supplementary Table 2: Primer sets used for qPCR and *in vitro* transcription, and oligonucleotide sequences of siRNAs used.**

| Primers |  |  |
| --- | --- | --- |
| (for qPCR) |  |  |
| Targets |  | Primer Sequences (5' - 3') |
| *TRA2β4* | forward | AGGAAAATGCGGAAGTCGTC |
|  | reverse | GACCGTGACCGGGTATAATG |
| *TRA2β1* | forward | CGGCGAGCGGGAATCCCG |
|  | reverse | GACCGTGACCGGGTATAATG |
| *GAPDH* | forward | AGCCACATCGCTCAGACAC |
|  | reverse | GCCCAATACGACCAAATCC |
| *NCL* | forward | GAAGGAAATGGCCAAACAGA |
|  | reverse | ACGCTTTCTCCAGGTCTTCA |
| *18S* | forward | CCCTATCAACTTTCGATGGTAGTCG |
|  | reverse | CCAATGGATCCTCGTTAAAGGATTT |
| *ACTB* | forward | ATTGCCGACAGGATGCAGA |
|  | reverse | GAGTACTTGCGCTCAGGAGGA |
| *pre-GAPDH* | reverse | CCCATACGACTGCAAAGACC |
|  |  |  |
| (*in vitro* transcription for biotinylated RNA pull-down assays) | | |
| *TRA2β4* | forward | [T7] ATGAGCGACAGCGGCGAGCAG |
|  | reverse | TTAATAGCGACGAGGTGAGTA |
| *TRA2β1* | forward | [T7] ATGAGCGACAGCGGCGAGCAG |
|  | reverse | TTAATAGCGACGAGGTGAGTA |
| *GAPDH* | forward | [T7] CCTCAACGACCACTTTGTCA |
|  | reverse | GGTTGAGCACAGGGTACTTTATT |
| *exon 2 F1* | forward | [T7] GTTAATGTTGAAGAAGGAAAATGC |
|  | reverse | ACTTCTGGTCTGATAATTAGC |
| *exon2 F2* | forward | [T7] CAGACCAGAAGTCCCACTTGT |
|  | reverse | TTAGCGTAGTGCTTTCTGAT |
| *exon2 F3* | forward | [T7] GCTAATTATCAGACCAGAAGT |
|  | reverse | CATCTTCCCCACTTCACACA |
| *exon2 F4* | forward | [T7] TGTGTGAAGTGGGGAAGATG |
|  | reverse | TTTCTTTTCTTCATTCTTCC |
| *exon2 F5* | forward | [T7] AAAAGAAATAAAAATGAAGTTAA |
|  | reverse | TTAGCGTAGTGCTTTCTGAT |
|  | *[T7]: T7 (ctaatacgactcactatagggaga) promoter sequence | |
|  |  |  |
|  |  |  |
| (for *TRA2β4* cloning) | | |
| *TRA2β4* | forward | AAAAAGAATTCGTGCGGGACGCGCTGCAGCTGG |
|  | reverse | AAAAAAAGCTTTTAAGTCTTTACATTATATATTAA |
|  | *The amplified fragment was subcloned into pCMV-FLAG (Agilent) using ecoRI and HindIII sites (these sequences are underlined) | |
|  |  |  |
| Sequence of siRNAs | |  |
| Name |  | Sequence (5'-3') |
| *TRA2β4* #1 siRNA  *TRA2β4* #2 siRNA |  | CUUGUAGAAUAUUGAGCAA  CUACGCUAAGAAUCCCGUU |
| *NCL* #1 siRNA |  | Santa Cruz Biotech. (sc-29230) |
| *NCL* #2 siRNA |  | GAGCUAACCCUUAUCUGUA (3’ UTR) |
